# Inpatient Oral Anticoagulation Management by Clinical Pharmacists: Safety and Cost effectiveness

**DOI:** 10.4021/jocmr2010.03.283w

**Published:** 2010-03-31

**Authors:** Sharath R. Hosmane, Johanna Tucker, Dave Osman, Steve Williams, Paul Waterworth

**Affiliations:** a Department of Cardiothoracic Surgery, University Hospital of South Manchester, Wythenshawe, Manchester, UK; b Pharmacy Department, University Hospital of South Manchester, Wythenshawe, Manchester, UK

## Abstract

**Background:**

Warfarin prescription for anticoagulation after cardiac surgery has always been a challenge for junior medical staff.

**Methods:**

A prospective study was carried out to assess the quality of anticoagulation control by junior doctors compared with clinical pharmacists at South Manchester University hospitals NHS Trust. The junior medical staff prescribed warfarin for 50 consecutive patients from April to September 2006 (group A, n = 50) and experienced clinical pharmacists dosed 46 consecutive patients between February and May 2007 (group B, n = 46).

**Results:**

In group A, 9 (18%) patients discharge was delayed because of lack of attainment of therapeutic International Normalised Ratio (INR) compared to 3 (6.5%) in group B. The total number of bed days resulting from the delay in group A was 21 compared to 4 in group B. Extrapolated over a year this would amount to approximately £15,750 extra cost incurred in group A opposed to £3000 in group B.

**Conclusions:**

The pharmacists were significantly better than junior doctors in achieving therapeutic INR, resulting in fewer discharge delays. The clinical pharmacists with experience in outpatient anticoagulation clinic can play an important role in inpatient oral anticoagulation management in post cardiac surgery patients thereby providing improved cost effective quality of care.

**Keywords:**

Warfarin; Pharmacist; Management

## Introduction

The safe management of oral anticoagulation in patients is a challenge in both ward and community. Sub-therapeutic and supra-therapeutic INR (International Normalised Ratio) can be hazardous to the patient. National Patient Safety Agency (NPSA) alert 18 was recently published to ensure safer prescribing, dispensing and administration of anticoagulants. We designed a study to assess the safety of anticoagulation management in cardiothoracic ward by clinical pharmacists with experience in anticoagulation clinics. This study was to assess the safety and efficiency of inpatient oral anticoagulation management by experienced clinical pharmacists compared with junior doctors following cardiac surgery.

## Patients and Methods

A prospective study was carried out to assess the quality of anticoagulation control by junior doctors compared with clinical pharmacists at South Manchester University hospitals NHS Trust. The junior medical staff prescribed warfarin for 50 consecutive patients from April to September 2006 (group A, n = 50) and experienced clinical pharmacists dosed 46 consecutive patients between Feb and May 2007 (group B, n = 46). There were six senior house officers in the department and the oncall doctor would do the prescribing of warfarin for all the patients on the given day. In the pharmacist group the prescribing was shared by two clinical pharmacists with experience in managing out patient anticoagulation clinic.

## Results

**Figure 1.**
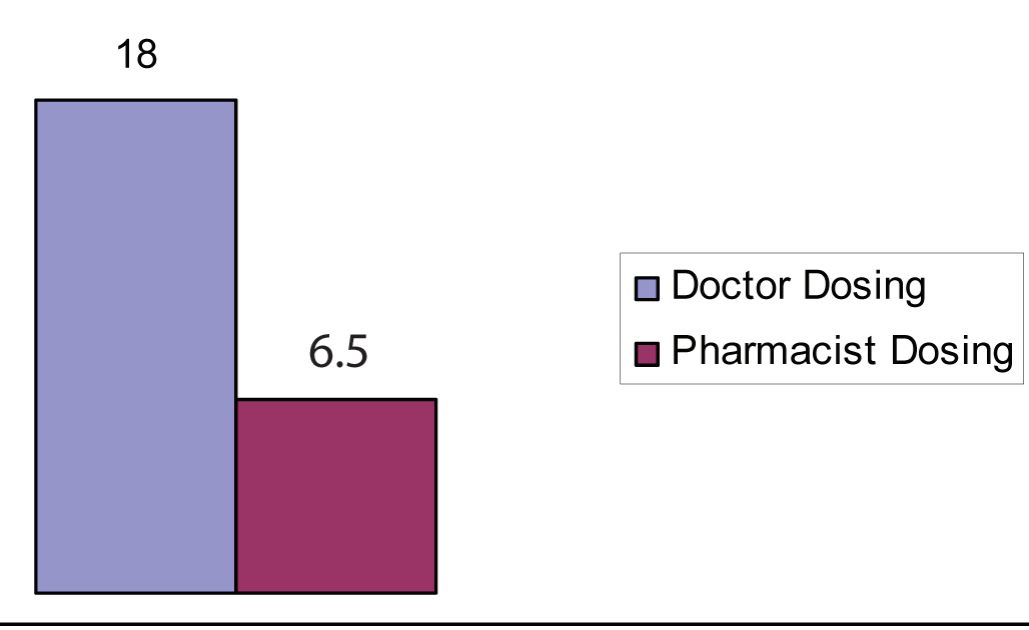
Percentage of patients with delayed discharge due to lack of therapeutic INR.

**Figure 2.**
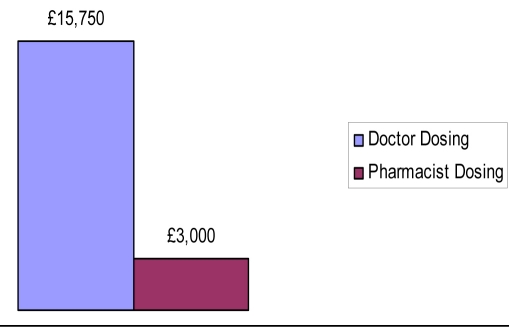
Comparative cost incurred due to extra bed days (data extrapolated to one year).

**Figure 3.**
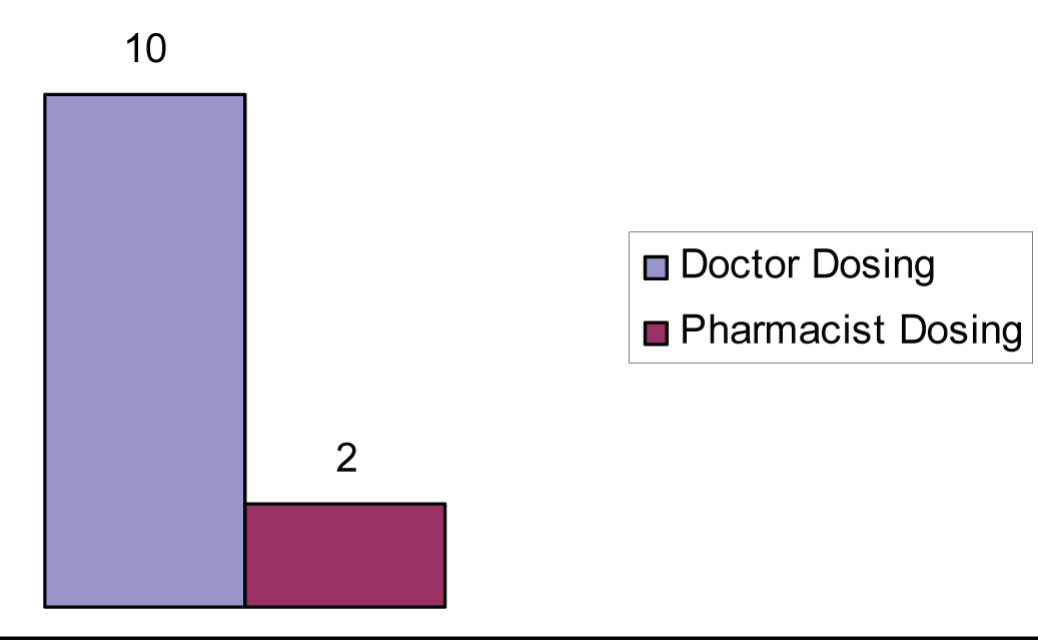
Percentage of patients with an INR greater than 5.0.

In group A, 10 patients INR was above 5 where as only 2 patients attained high INR in group B. In group A, 9 (18%) patients discharge was delayed because of lack of attainment of therapeutic INR compared to 3 (6.5%) in group B. The total number of bed days resulting from the delay in group A was 21 compared to 4 in group B. Extrapolated over a year this would amount to approximately £15,750 extra cost incurred in group A opposed to £3000 in group B, Figure 1, 2, 3.

## Discussion

In our pilot study the pharmacists were significantly better than junior doctors in achieving therapeutic INR, resulting in fewer discharge delays. The clinical pharmacists with experience in out patient anticoagulation clinic can play an important role in inpatient oral anticoagulation management in post cardiac surgery patients thereby providing improved cost effective quality of care. The junior doctors in the cardiothoracic department are more familiar than junior doctors in other specialties with warfarin prescription because of the patient population. Despite this there was significant difference compared with pharmacist management. Therefore the difference is likely to be greater if the study had included junior doctors in other specialties where warfarin use is less common. The results of our study are of interest in keeping with the recently published National Patient Safety Agency (NPSA) alert 18 [1] which identifies anticoagulants as one of four high risk medications that require multidisciplinary interventions to reduce the harm to patients in hospitals.

Warfarin administration is a challenge to the junior medical staff. The challenge is to attain the therapeutic range of INR with lowest effective dose. The interaction of warfarin with several other commonly used medications and variability in response from different patients makes it very difficult to attain and maintain therapeutic range of INR. The lower than normal INR would predispose the patients to thrombotic risk and higher than therapeutic range expose the patients to potentially life threatening bleeding complications. Landefeld et al, in their meta-analysis showed warfarin administration is associated with a 0.6% annual risk of bleeding-related death, a 3% annual risk of a major bleeding event, and a 9.6% annual risk of a major or minor bleeding event, with the risk being highest at the start of therapy [2].

Several other studies have showed the effectiveness of clinical pharmacists in out patient [3-7] and inpatient [8, 9] anticoagulation management. Although the out patient service is largely run by clinical pharmacists in UK, their role in inpatient anticoagulation is limited. Based on our study and several other studies in the literature it appears that clinical pharmacists anticoagulation management is safer for patients and more cost effective for hospitals.

## References

[1] National Patient Safety Agency anticoagulation guidelines (alert 18).

[2] Landefeld CS, Beyth RJ (1993). Anticoagulant-related bleeding: clinical epidemiology, prediction, and prevention. Am J Med.

[3] Witt DM, Sadler MA, Shanahan RL, Mazzoli G, Tillman DJ (2005). Effect of a centralized clinical pharmacy anticoagulation service on the outcomes of anticoagulation therapy. Chest.

[4] Chiquette E, Amato MG, Bussey HI (1998). Comparison of an anticoagulation clinic with usual medical care: anticoagulation control, patient outcomes, and health care costs. Arch Intern Med.

[5] Locke C, Ravnan SL, Patel R, Uchizono JA (2005). Reduction in warfarin adverse events requiring patient hospitalization after implementation of a pharmacist-managed anticoagulation service. Pharmacotherapy.

[6] Wilt VM, Gums JG, Ahmed OI, Moore LM (1995). Outcome analysis of a pharmacist-managed anticoagulation service. Pharmacotherapy.

[7] Garabedian-Ruffalo SM, Gray DR, Sax MJ, Ruffalo RL (1985). Retrospective evaluation of a pharmacist-managed warfarin anticoagulation clinic. Am J Hosp Pharm.

[8] Bond CA, Raehl CL (2004). Pharmacist-provided anticoagulation management in United States hospitals: death rates, length of stay, Medicare charges, bleeding complications, and transfusions. Pharmacotherapy.

[9] Damaske DL, Baird RW (2005). Development and implementation of a pharmacist-managed inpatient warfarin protocol. Proc (Bayl Univ Med Cent).

